# Association of sweetened beverage intake with incident risk of breast cancer: a prospective cohort study

**DOI:** 10.3389/fnut.2025.1680542

**Published:** 2026-01-14

**Authors:** Jiayi Huang, Fuhua Wu, Weiwei Chen, Ding Ye, Yiping Tian, Jiayu Li, Jing Guo, Jianming Wang, Yingying Mao, Xiaohui Sun

**Affiliations:** 1 Department of Epidemiology, School of Public Health, Zhejiang Chinese Medical University, Hangzhou, China; 2 Disease Control and Supervision Section, Jiashan County Dayun Town Health Center, Jiaxing, China; 3 Department of Epidemiology, Center for Global Health, School of Public Health, Nanjing Medical University, Nanjing, China; 4 Department of Pathology, Zhejiang Cancer Hospital, Hangzhou Institute of Medicine (HIM), Chinese Academy of Sciences, Hangzhou, Zhejiang, China; 5 Hangzhou Institute of Medicine, Chinese Academy of Sciences, Hangzhou, China

**Keywords:** artificially sweetened beverages, breast cancer, cohort study, pure fruit/vegetable juice, sugar-sweetened beverages

## Abstract

**Background:**

This large prospective cohort study aimed to clarify uncertain associations between sugar-sweetened beverages (SSB), artificially sweetened beverages (ASB), pure fruit/vegetable juice and breast cancer risk.

**Methods:**

In 86,247 cancer-free UK Biobank participants at baseline, Cox proportional hazard models were performed to investigate the associations of SSB, ASB, pure fruit/vegetable juice with incident breast cancer risk.

**Results:**

In total, 2,644 cases of incident breast cancer occurred during the median follow-up of 10 years. In Cox proportional hazards model, participants consuming >0 and ≤1 serving/day of pure fruit/vegetable juice had a hazard ratio (HR) of 1.13 (95% CI: 1.05–1.23; *P* = 0.002) compared to non-consumers (*P* = 0.004 for trend). However, no associations were observed for SSB and ASB with breast cancer risk. We also found that replacing 1 serving per day of pure fruit/vegetable juice with ASB was associated with 10% lower risk of incident breast cancer in substitution analyses.

**Conclusions:**

Our findings indicated that pure fruit/vegetable juice was associated with increased risk of breast cancer, highlighting the potential role of sweetened beverages in breast cancer prevention strategies and call for further research to understand the underlying mechanisms.

## Introduction

Breast cancer is the most common cancer diagnosed among women globally (1). There were about 2.3 million new cases of breast cancer in 2022, accounting for 11.6% of all new cases of malignant tumors in women (2). Several risk factors, such as age, genetic predisposition, family history, hormonal exposure and lifestyle factors, have been identified for breast cancer (3). Diet, as an important lifestyle factor, is estimated to contribute to approximately 30% of cancer cases in developed countries (4), with healthy dietary patterns shown to reduce breast cancer risk by 11% (5). Recently, high-sugar dietary patterns have gained attention as a potential contributor to breast cancer.

Sugar-sweetened beverages (SSB) are a major source of added sugars, with their consumption rising globally (6). Between 2011 and 2014, U.S. adults consumed an average of approximately 145 kcal per day from SSB (7). Artificially sweetened beverages (ASB) and pure fruit/vegetable juice are also being promoted as alternatives to meet consumer demand for healthier beverages. It was not until recently that role of them on breast cancer risk began to receive attention, but yielding largely inconsistent results (8–10). In a large French cohort involving 101,257 participants (8), the consumption of SSB was found to be positively associated with an increased risk of breast cancer. Similar findings were reported in a cohort study conducted in Melbourne (9). In contrast, a longitudinal study of Framingham offspring cohort found no association of SSB and breast cancer risk (10). For ASB, both the French cohort (8) and the Melbourne cohort (9) found no association with breast cancer. However, another cohort study suggested that ASB might be linked to an increased risk of breast cancer (11). Even pure fruit/vegetable juice, such as fruit juices, were also reported to be associated with increased risk of developing breast cancer (12). However, several studies reported no significant association between pure fruit/vegetable juice consumption and breast cancer risk (8, 13–16). Given the limited sample size, inadequate adjustment for confounding factors, and inconclusive findings in previous studies, further research is needed to clarify these associations.

Herein, in the present study, we aimed to investigate the associations between intakes of SSB, ASB and pure fruit/vegetable juice and risk of breast cancer, and the effect of substituting beverage types on this association using a large population-based cohort.

## Method and materials

### Study design

This study was based on the UK Biobank, a prospective population-based cohort study that has recruited over 500,000 participants between 2006 and 2010 (17). Participants attended one of 22 assessment centers across England, Wales and Scotland and provided sociodemographic and lifestyle information via a self-completed and touch-screen questionnaire (18). UK Biobank study approval was obtained from the National Health Service and the National Research Ethics Service and was renewed (18). The UK Biobank received ethical approval from the North West-Haydock Research Ethics Committee. All participants in this study provided informed consent when they were recruited. This research has been conducted using the UK Biobank Resource under Application Number 99685.

**Figure 1:**
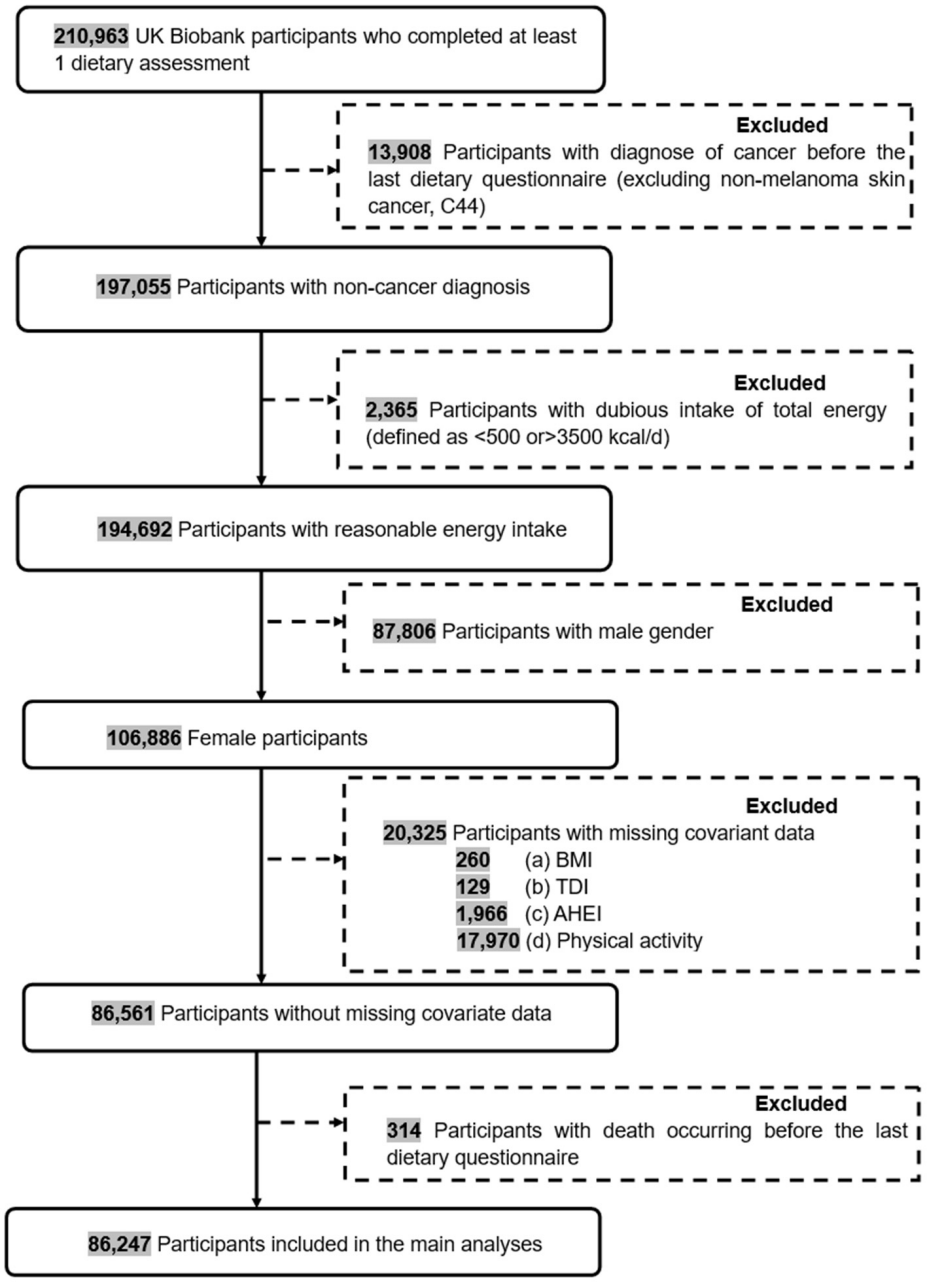
The flowchart of inclusion and exclusion of study participants in the present study.

We only included female individuals who attended at least one occasion of the online 24-h recall assessment in the present study. The exclusion criteria were: (1) participants with diagnose of cancer before baseline (excluding non-melanoma skin cancer, C44); (2) individuals reporting abnormal energy intake (defined as energy intake of < 500 or >3500 kcal/d); (3) individuals who had missing data for covariates, such as body mass index (BMI), Townsend deprivation index (TDI), Alternative Healthy Eating Index (AHEI) and physical activity; (4) individuals with death occurring before the last dietary questionnaire. The final data set consists of 86,247 individuals (Figure 1).

### Assessment of beverage consumption

In UK Biobank, dietary information was collected via the Oxford WebQ, a web-based 24-h dietary assessment tool that was developed specifically for use in large population studies and has been validated against an interviewer administered (19). The dietary assessments were conducted as follows: (1) from April 2009 to September 2010; (2) from February 2011 to April 2011; (3) from June 2011 to September 2011; (4) from October 2011 to December 2011; (5) from April 2012 to June 2012 (20). Of the 86,247 participants in our analysis, 38% completed the dietary assessments once, 23% twice, 21% three times, 15% four times, and 3% five times. Daily beverage intake was assessed by following question: “How many glasses, cans, or cartons containing 250 mL of sugar-sweetened beverages, artificially sweetened beverages or pure fruit/vegetable juices did you drink yesterday?”. Beverage types were categorized as follows: carbonated drinks, fizzy drinks, and squash were classified as SSB; low-calorie drinks as ASB and pure grapefruit juice, pure orange juice, and other pure fruit/vegetable juices as pure fruit/vegetable juices (21). Notably, the category of pure fruit/vegetable juice was exclusively 100% fruit- or vegetable-derived beverages without additives, and was explicitly distinguished from concentrated juices (e.g., squash/cordial), smoothies, and fruit drinks. Beverage intake values were standardized to 1 serving (250 mL) and classified into 0 serving/day, >0 and ≤ 1 serving/day and >1 serving/day (20). In the present study, the mean beverage intake was calculated for participants who attended on more than one occasion.

### Assessment of covariates

The covariates in the present study were selected following prior epidemiological studies (21, 22). Information on sociodemographic and lifestyle behaviors were ascertained at baseline using a self-reported online questionnaire, which included age (continuous in years), ethnicity (White, Asian or Asian British, Black or Black British, mixed or other), education qualifications (college or university degree, vocational qualifications, optional national exams at ages 17–18 years, national exams at age 16 years, none of the above, unknown), TDI (continuous), physical activity (continuous in MET-hours/week), smoking status (never, previous, current, unknow), drinking status (never, previous, current, unknow), BMI (continuous in kg/m^2^), hypertension (no, yes), type 2 diabetes (no, yes), major cardiovascular disease (no, yes), family history of breast cancer (no, yes), total energy intake (continuous in kcal), AHEI (continuous), total sugar intake (continuous in g), menopausal status (post-menopausal, premenopausal), parity (nulliparous, one to two, three or more, unknown), hormone replacement therapy (never, past, current, unknown) and oral contraceptive pill use (never, past, current, unknown).

The area-based TDI was used to assess socioeconomic status, which was derived from consensus data on employment, housing, car ownership, and household overcrowding, corresponding to the postcode of residence (22, 23). Physical activity was measured as metabolic equivalents using a questionnaire based on the International Physical Activity Questionnaire (IPAQ), which were calculated by walking, moderate and vigorous activity per week by the weights of 2.5, 4, and 8, and then summing them. BMI was calculated by dividing the weight (kg) by height squared (m^2^). The International Statistical Classification of Diseases and Related Health Problems *10*th Revision (*ICD*-*10*) was used to identify the chronic diseases, such as type 2 diabetes (E10-E14), hypertension (I10-I13, I15, and O10) and major cardiovascular diseases [including myocardial infarction (I21-I23, I24.1, and I25.2) and stroke (I60, I61, I63, and I64)]. The total sugar and energy intake were provided by the UK Biobank, which calculated based on McCance and Widdowson's “The composition of food” (24). Since the Healthy Eating Index (HEI) (25) was primarily designed to assess overall diet quality in relation to major chronic diseases, we used a simplified 5-component AHEI score, developed by Anderson et al. (21), as an alternative to HEI (26). It was calculated based on five dietary factors (mean intake of fat, fruit, vegetables, red meat and processed meat intake) according to the scoring system and ranged from 0 to 50.

### Ascertainment of outcome

The incident breast cancer cases were identified using ICD-10 code (C50) from cancer registry data, supplemented by record-level hospital inpatient data and death registry data (27). The follow-up time for each participant was calculated as the number of years from the date of everyone completed the dietary assessment until the earlies of the following: date of first breast cancer diagnosis, date of death, date of loss to follow-up, or last date of follow-up available data (31 October 2022 for England, 31 May 2022 for Wales, and 31 August 2022 for Scotland).

### Statistical analysis

Baseline characteristics were examined according to the three types of beverages. Continuous variables and categorical variables were expressed as means (SDs) and numbers (percentage), separately. Comparisons among beverage intake were performed using Student's *t*-tests or Wilcoxon tests for continuous variables, and the Chi-square tests for categorical variables. Associations of SSB, ASB, pure fruit/vegetable juice with breast cancer risk were estimated using Cox proportional hazard models and are presented as hazard ratios (HRs) and 95% confidence interval (CI). Three models were conducted in our analyses. Model 1 was adjusted for age. Model 2 was additionally adjusted for ethnicity, education qualification, TDI, physical activity, smoking status, drinking status, BMI, hypertension, type 2 diabetes, cardiovascular disease and family history of breast cancer, and the fully adjusted model (Model 3) was additionally adjusted for total energy intake, AHEI score, total sugar intake, menopausal status, parity, hormone replacement therapy and oral contraceptive pill use.

To assess the robustness of our results, several sensitivity analyses were conducted. We first excluded the first 2 years of follow-up to prevent the reverse causality. To evaluate the specific association of each beverage, we also excluded individuals who reported consuming other types of beverages and reperformed analysis. Additionally, we re-ran the analyses after excluding the participants reporting weekly changes in their diet. Moreover, a substitution analysis was performed to evaluate the effect of replacing one beverage with another. In this approach, the substitution model estimates the beta coefficient by including both beverage variables in the regression model. The effect of substituting one beverage for another can then be derived through mathematical modeling (28).

Additionally, we evaluated whether the associations between beverages and breast cancer risk were modified by age (< 60 and ≥60 years), BMI (< 25, 25–30, ≥30 kg/m^2^), physical activity (low, moderate, high), menopausal status (premenopausal, post-menopausal) and diabetes (yes or no) using multivariate models to test for multiplicative interactions.

We considered statistical significance as a 2-sided *P* < 0.05. Statistical analyses were performed using R, version 4.1.1.

## Results

The baseline characteristics of participants according to the consumption of the different beverages are shown in the Table 1. Of the 86,247 female participants included the mean (SD) age was 55.1 (7.9). Among them, 29,132 (33.8%) reported consuming SSB, 19,319 (22.4%) consumed ASB, and 43,081 (50.0%) consumed pure fruit/vegetable juice.

**Table 1:** Baseline characteristics of participants by category of beverage intake.

**Characteristics^a^**	**Sugar-sweetened beverages**				**Artificially sweetened beverages**				**Pure fruit/vegetable juice**			
	**0 serving/d (*****n*** = **57,115, 66.2%)**	>**0–1 serving/d (*****n*** =**2 0,835, 24.2%)**	>**1 serving/d (*****n*** =**8,297, 9.6%)**	***P*****-value**	**0 serving/d (*****n*** =**6 6,928, 77.6%)**	>**0–1 serving/d (*****n*** = **9,300, 10.8%)**	>**1 serving/d (*****n*** =**1 0,019, 11.6%)**	***P*****-value**	**0 serving/d (*****n*** =**4 3,166, 50.0%)**	>**0–1 serving/d (*****n*** = **37,636, 43.6%)**	>**1 serving/d (*****n*** = **5,445, 6.3%)**	***P*****-value**
Age, median (IQR)	56.0 (13.0)	55.0 (13.0)	52.0 (14.0)	< 0.001	56.0 (13.0)	55.0 (13.0)	52.0 (13.0)	< 0.001	55.0 (13.0)	56.0 (13.0)	56.0 (13.0)	< 0.001
Ethnicity, *n* (%)				< 0.001				< 0.001				< 0.001
Asian or Asian British	867 (1.52)	317 (1.52)	131 (1.58)		1,135 (1.70)	105 (1.13)	75 (0.75)		767 (1.78)	478 (1.27)	70 (1.29)	
Black or Black British	585 (1.02)	278 (1.33)	245 (2.95)		892 (1.33)	87 (0.94)	129 (1.29)		566 (1.31)	377 (1.00)	165 (3.03)	
White	54,803 (96.0)	19,855 (95.3)	7735 (93.2)		63,749 (95.3)	8,973 (96.5)	9,671 (96.5)		41,119 (95.3)	36,224 (96.2)	5,050 (92.7)	
Mixed or other	755 (1.32)	346 (1.66)	164 (1.98)		10,22 (1.53)	120 (1.29)	123 (1.23)		626 (1.45)	498 (1.32)	141 (2.59)	
Unknown	105 (0.18)	39 (0.19)	22 (0.27)		130 (0.19)	15 (0.16)	21 (0.21)		88 (0.20)	59 (0.16)	19 (0.35)	
Education qualification, *n* (%)				< 0.001				< 0.001				< 0.001
A levels/AS levels	8,161 (14.3)	3,024 (14.5)	1,233 (14.9)		9,486 (14.2)	1,446 (15.5)	1,486 (14.8)		6,204 (14.4)	5,446 (14.5)	768 (14.1)	
College or University degree	2,4762 (43.4)	9,551 (45.8)	3,258 (39.3)		29,995 (44.8)	3,930 (42.3)	3,646 (36.4)		16,826 (39.0)	17,926 (47.6)	2,819 (51.8)	
O levels/GCSEs/CSEs	1,4907 (26.1)	5,235 (25.1)	2,432 (29.3)		16,924 (25.3)	2,537 (27.3)	3,113 (31.1)		12,426 (28.8)	9,004 (23.9)	1,144 (21.0)	
Vocational qualifications	4,824 (8.45)	1,741 (8.36)	779 (9.39)		5,566 (8.32)	795 (8.55)	983 (9.81)		3,920 (9.08)	3,015 (8.01)	409 (7.51)	
Other	4,279 (7.49)	1,240 (5.95)	568 (6.85)		4,759 (7.11)	564 (6.06)	764 (7.63)		3,644 (8.44)	2,151 (5.72)	292 (5.36)	
Unknown	182 (0.32)	44 (0.21)	27 (0.33)		198 (0.30)	28 (0.30)	27 (0.27)		146 (0.34)	94 (0.25)	13 (0.24)	
Townsend deprivation index, median (IQR)	−2.3 (3.8)	−2.3 (3.8)	−2.0 (4.1)	< 0.001	−2.3 (3.8)	−2.4 (3.6)	−2.2 (3.9)	< 0.001	−2.2 (3.9)	−2.4 (3.7)	−2.2 (4.1)	< 0.001
BMI, median (IQR)	25.4 (5.7)	25.3 (5.7)	26.0 (6.6)	< 0.001	25.0 (5.3)	26.3 (6.2)	27.4 (7.2)	< 0.001	25.8 (6.0)	25.1 (5.5)	25.0 (5.6)	< 0.001
Physical activity, MET-h/wk, median (IQR)	120.0 (191.5)	116.0 (187.8)	115.0 (191.6)	0.075	120.0 (191.0)	115.0 (190.9)	113.0 (191.3)	< 0.001	116.0 (193.1)	121.0 (186.3)	126.0 (200.1)	< 0.001
Smoking status, *n* (%)				< 0.001				< 0.001				< 0.001
Currently	3,821 (6.70)	1,196 (5.74)	673 (8.11)		4,343 (6.49)	554 (5.96)	793 (7.91)		3,405 (7.89)	1,955 (5.19)	330 (6.06)	
Never	34,124 (59.7)	13,187 (63.3)	5,058 (61.0)		41,033 (61.3)	5,541 (59.6)	5,795 (57.8)		24,877 (57.6)	23,922 (63.6)	3,570 (65.6)	
Previous	19,078 (33.4)	6,424 (30.8)	2,552 (30.8)		21,457 (32.1)	3,188 (34.3)	3,409 (34.0)		14,805 (34.3)	11,713 (31.1)	1,536 (28.2)	
Unknown	92 (0.16)	28 (0.13)	14 (0.17)		95 (0.14)	17 (0.18)	22 (0.22)		79 (0.18)	46 (0.12)	9 (0.17)	
Drinking status, *n* (%)				< 0.001				< 0.001				< 0.001
Currently	53,373 (93.4)	19,312 (92.7)	7,543 (90.9)		62,261 (93.0)	8,754 (94.1)	9,213 (92.0)		39,847 (92.3)	35,397 (94.1)	4,984 (91.5)	
Never	2,103 (3.68)	887 (4.26)	432 (5.21)		2,709 (4.05)	299 (3.22)	414 (4.13)		1,842 (4.27)	1,310 (3.48)	270 (4.96)	
Previous	1,616 (2.83)	627 (3.01)	318 (3.83)		1,931 (2.89)	242 (2.60)	388 (3.87)		1,458 (3.38)	916 (2.43)	187 (3.43)	
Unknown	23 (0.04)	9 (0.04)	4 (0.05)		27 (0.04)	5 (0.05)	4 (0.04)		19 (0.04)	13 (0.03)	4 (0.07)	
Diabetes status, *n* (%)				< 0.001				< 0.001				< 0.001
No	54,864 (96.1)	20,127 (96.6)	7,930 (95.6)		64,680 (96.6)	8,891 (95.6)	9,350 (93.3)		41,232 (95.5)	36,427 (96.8)	5,262 (96.6)	
Yes	2,251 (3.94)	708 (3.40)	367 (4.42)		2,248 (3.36)	409 (4.40)	669 (6.68)		1,934 (4.48)	1,209 (3.21)	183 (3.36)	
Cardiovascular disease status, *n* (%)				0.358				0.397				0.011
No	56,640 (99.2)	20,674 (99.2)	8,219 (99.1)		66,387 (99.2)	9,221 (99.2)	9,925 (99.1)		42,769 (99.1)	37,361 (99.3)	5,403 (99.2)	
Yes	475 (0.83)	161 (0.77)	78 (0.94)		541 (0.81)	79 (0.85)	94 (0.94)		397 (0.92)	275 (0.73)	42 (0.77)	
Hypertension status, *n* (%)				< 0.001				< 0.001				< 0.001
No	52,498 (91.9)	19,326 (92.8)	7,630 (91.6)		61,865 (92.4)	8,560 (92.0)	9,001 (89.8)		39,505 (91.5)	34,884 (92.7)	5,037 (92.5)	
Yes	4,617 (8.08)	1,509 (7.24)	695 (8.38)		5,063 (7.56)	740 (7.96)	1,018 (10.2)		3,661 (8.48)	2,752 (7.31)	408 (7.49)	
Family history of breast cancer, *n* (%)				0.690				0.157				0.620
No	39,585 (69.3)	14,471 (69.5)	5,868 (70.7)		46,451 (69.4)	6,477 (69.6)	6,996 (69.8)		29,862 (69.2)	26,289 (69.9)	3,773 (69.3)	
Yes	17,530 (30.7)	6,364 (24.2)	2,429 (29.3)		20,477 (30.6)	2,823 (30.4)	3,023 (30.2)		13,304 (30.8)	11,347 (30.1)	1,672 (30.7)	
AHEI score, mean (SD)^b^	29.0 (10.5)	27.6 (10.6)	26.9 (10.8)	< 0.001	28.5 (10.6)	28.2 (10.5)	28.0 (10.6)	< 0.001	28.6 (10.7)	28.2 (10.5)	28.9 (10.5)	< 0.001
Total energy intake, g/d, mean (SD)	1,863 (480)	1,958 (443)	2,065 (506)	< 0.001	1,907 (480)	1,909 (433)	1,891 (507)	0.006	1,838 (491)	1,957 (451)	2,081 (475)	< 0.001
Total sugar intake, mean (SD)	111 (42.3)	125 (39.5)	153 (48.7)	< 0.001	119 (44.2)	118 (39.8)	117 (47.4)	< 0.001	108 (43.1)	125 (40.0)	157 (48.1)	< 0.001
Menopausal status, *n* (%)				< 0.001				< 0.001				< 0.001
Post-menopausal	36,718 (64.3)	12,774 (61.3)	4,326 (52.1)		43,205 (64.6)	5,466 (58.8)	5,147 (51.4)		26,564 (61.5)	23,921 (63.6)	3,333 (61.2)	
Premenopausal	20,378 (35.7)	8,052 (38.6)	3,963 (47.8)		23,696 (35.4)	3,831 (41.2)	4,866 (48.6)		16,578 (38.4)	13,705 (36.4)	2,110 (38.8)	
Unknown	19 (0.03)	9 (0.04)	8 (0.10)		27 (0.04)	3 (0.03)	6 (0.06)		24 (0.06)	10 (0.03)	2 (0.04)	
Parity, *n* (%)				< 0.001				< 0.001				0.001
Nulliparous	12,570 (22.0)	4,814 (23.1)	2,045 (24.6)		14,992 (22.4)	2,110 (22.7)	2,327 (23.2)		9,583 (22.2)	8,570 (22.8)	1,276 (23.4)	
One to two	32,292 (56.5)	11,740 (56.3)	4,580 (55.2)		37,595 (56.2)	5,331 (57.3)	5,686 (56.8)		24,268 (56.2)	21,338 (56.7)	3,006 (55.2)	
Three or more	12,228 (21.4)	4,274 (20.5)	1,668 (20.1)		14,314 (21.4)	1,856 (20.0)	2,000 (20.0)		9,303 (21.6)	7,709 (20.5)	1,158 (21.3)	
Unknown	25 (0.04)	7 (0.03)	4 (0.05)		27 (0.04)	3 (0.03)	6 (0.06)		12 (0.03)	19 (0.05)	5 (0.09)	
Oral contraceptive pill use, *n* (%)				< 0.001				< 0.001				0.038
Current	602 (1.05)	268 (1.29)	124 (1.49)		708 (1.06)	128 (1.38)	158 (1.58)		499 (1.16)	437 (1.16)	58 (1.07)	
Never	8,354 (14.6)	2,925 (14.0)	1,133 (13.7)		10,131 (15.1)	1,150 (12.4)	1,131 (11.3)		6,156 (14.3)	5,391 (14.3)	865 (15.9)	
Past	4,8090 (84.2)	17,612 (84.5)	7,020 (84.6)		55,995 (83.7)	8,012 (86.2)	8,715 (87.0)		36,49 (84.4)	31,762 (84.4)	4,511 (82.8)	
Unknown	69 (0.12)	30 (0.14)	20 (0.24)		94 (0.14)	10 (0.11)	15 (0.15)		62 (0.14)	46 (0.12)	11 (0.20)	
Hormone replacement therapy, *n* (%)				< 0.001				< 0.001				< 0.001
Current	1,071 (1.88)	368 (1.77)	171 (2.06)		1,235 (1.85)	162 (1.74)	213 (2.13)		861 (1.99)	672 (1.79)	77 (1.41)	
Never	36,875 (64.5)	13,796 (66.2)	5,756 (69.4)		43,583 (65.1)	6,076 (65.3)	6,768 (67.6)		28,150 (65.2)	24,582 (65.3)	3,695 (67.9)	
Past	19,068 (33.4)	6,632 (31.8)	2,350 (28.3)		21,982 (32.8)	3,045 (32.7)	3,023 (30.2)		14,073 (32.6)	12,321 (32.7)	1,656 (30.4)	
Unknown	101 (0.18)	39 (0.19)	20 (0.24)		128 (0.19)	17 (0.18)	15 (0.15)		82 (0.19)	61 (0.16)	17 (0.31)	

IQR, interquartile range; BMI, body mass index (calculated as weight in kilograms divided by height in meters squared); MET, metabolic equivalent; AHEI, Alternative Healthy Eating Index; SD, standard deviation. ^a^The values for categorical variables are given as numbers (percentage) and values for continuous variables are given as mean (standard deviation). ^b^AHEI included five items, including red meat, processed meat, fruit, vegetables and fat.Each dietary item scored 0 (unhealthiest), 5 and 10 (healthiest).

Participants with higher intake of SSB were younger, had a higher BMI and lower AHEI score, also consumed more total energy and total sugar intake compared with participants with lower SSB intake (all *P* < 0.001). Participants with a higher consumption of ASB were younger, also had a higher BMI and lower AHEI score compared with participants with a lower consumption of ASB (all *P* < 0.001). However, compared with participants who did not consume ASB, participants with higher consumption of ASB showed a slightly decreased energy intake (*P* = 0.006) and total sugar intake (*P* < 0.001). Participants consuming pure fruit/vegetable juice more often were older (*P* < 0.001), had a lower BMI and higher AHEI score, but still had higher total energy and sugar intake than participants with lower intakes of pure fruit/vegetable juice (all *P* < 0.001).

During the median follow-up of 10.0 years, 2,644 incident breast cancer cases, 1,959 deaths and 192 losses occurred. Consumptions of pure fruit/vegetable juice was found significantly associated with the risk of incident breast cancer, whereas no such association was observed for SSB or ASB.

In the fully adjusted model (Model 3), the HR for participants with >0 and ≤ 1 serving/day of pure fruit/vegetable juice was 1.13 (95% CI: 1.05–1.23; *P* = 0.002), compared with participants who did not consume pure fruit/vegetable juice (*P* = 0.004 for trend) (Table 2). After excluding the first 2 years of follow-up, there still was significant association between the consumption of pure fruit/vegetable juice and breast cancer risk (HR: 1.14, 95% CI:1.04–1.25 for >0 and ≤ 1 serving/day; *P* = 0.005 for trend) in the fully adjusted model (Supplementary Table 1). Applying an exclusive consumer analytical strategy to isolate beverage-specific effects, our sensitivity analysis also identified a significant positive association for breast cancer risk in relation to pure fruit/vegetable juice (HR: 1.23, 95% CI:1.10–1.37 for >0 and ≤ 1 serving/day; *P* = 0.015 for trend) (Supplementary Table 2). Additionally, a significant association emerged for SSBs after excluding individuals who consumed other types of beverages (*P* for trend = 0.015) (Supplementary Table 2). Moreover, after excluding participants who self-reported weekly changes in daily diet, a significant association between pure fruit/vegetable juice consumption and breast cancer risk persisted in the fully adjusted model (HR: 1.15, 95% CI: 1.05–1.25 for >0 and ≤ 1 serving/day; *P* = 0.004 for trend) (Supplementary Table 3).

**Table 2:** The associations between three types of beverage intake and risk of incident breast cancer.

**Beverages**	**Incident cases/person years**	**Model 1**		**Model 2**		**Model 3**	
		**HR (95% CI)**	***P*** **-value**	**HR (95% CI)**	***P*** **-value**	**HR (95% CI)**	***P*** **-value**
**Sugar-sweetened beverages**							
0 serving/d	1,733/571468.28	Ref		Ref		Ref	
>0–1 serving/d	659/208290.35	1.06 (0.97–1.16)	0.232	1.07 (0.97–1.17)	0.163	1.07 (0.98–1.17)	0.139
>1 serving/d	252/83074.63	1.05 (0.92–1.20)	0.493	1.05 (0.92–1.20)	0.486	1.08 (0.94–1.24)	0.287
*P* for trend		0.259		0.215		0.120	
**Artificially sweetened beverages**							
0 serving/d	2,062/669356.68	Ref		Ref		Ref	
>0–1 serving/d	292/93081.13	1.04 (0.92–1.18)	0.515	1.02 (0.90–1.15)	0.764	1.02 (0.90–1.15)	0.777
>1 serving/d	290/100395.45	0.99 (0.87–1.12)	0.819	0.94 (0.83–1.07)	0.380	0.95 (0.83–1.07)	0.384
*P* for trend		0.982		0.495		0.496	
**Natural juices**							
0 serving/d	1,256/432239.42	Ref		Ref		Ref	
>0–1 serving/d	1,219/376146.33	1.10 (1.02–1.19)	0.014	1.12 (1.04–1.22)	0.004	1.13 (1.05–1.23)	0.002
>1 serving/d	169/54447.52	1.06 (0.91–1.25)	0.446	1.10 (0.93–1.29)	0.264	1.14 (0.97–1.35)	0.114
*P* for trend		0.039		0.011		0.004	

HR, hazard ratio; CI, confidence interval. Values are standardized to 1 serving (250 mL). Model 1 was adjusted for age. Model 2 was additionally adjusted for ethnicity, education qualification, Townsend deprivation index, physical activity, smoking status and drinking consumption status, body mass index, hypertension status, diabetes status, cardiovascular disease status and family history of breast cancer. Model 3 was additionally adjusted for total energy intake, total sugar intake and AHEI score, menopausal status, parity, hormone replacement therapy and oral contraceptive pill use.

In substitution analyses, we only observed that replacing 1 serving per day of pure fruit/vegetable juice with ASB was associated with 10% lower risk of incident breast cancer (HR: 0.90, 95%CI: 0.82–0.97, *P* = 0.010) (Table 3). However, there was no significant risk difference in incident breast cancer when replacing pure fruit/vegetable juice with SSB (HR: 0.94, 95% CI: 0.86–1.03, *P* = 0.180). Similarly, replacing 1 serving per day of ASB with SSB was no significant risk difference in incident breast cancer (HR: 1.05, 95% CI: 0.97–1.14, *P* = 0.223).

**Table 3:** Substitution analysis for the association between risk of incident breast cancer and beverage intake.

**Substitution analysis**	**Hypothetically decrease intake for each of these three beverages, hazard ratio (95% CI)**					
	**Sugar-sweetened beverages**		**Artificially sweetened beverages**		**Pure fruit/vegetable juice**	
	**HR (95% CI)**	***P*** **-value**	**HR (95% CI)**	***P*** **-value**	**HR (95% CI)**	***P*** **-value**
With sugar-sweetened beverages	Ref		1.05 (0.97, 1.14)	0.223	0.94 (0.86, 1.03)	0.180
With artificially sweetened beverages	0.95 (0.88, 1.03)	0.223	Ref		0.90 (0.82, 0.97)	0.010
With natural pure fruit/vegetable juice	1.06 (0.97, 1.16)	0.180	1.12 (1.03, 1.21)	0.010	Ref	

Ref, reference; HR, hazard ratio; CI, confidence interval. HR was calculated based on fully adjusted model, adjusted for age, ethnicity, education qualification, Townsend deprivation index, physical activity, smoking status, drinking status, BMI, hypertension status, diabetes status, cardiovascular disease status and family history of breast cancer, total energy intake, total sugar intake, AHEI score, menopausal status, parity, hormone replacement therapy and oral contraceptive pill use.

We finally assessed the interactions between three different types of beverage intake and age, BMI, type 2 diabetes, physical activity, and menopausal status on the risk of breast cancer. There were no significant interactions between these subgroup factors and SSB (Supplementary Table 4), ASB (Supplementary Table 5) and pure fruit/vegetable juice (Supplementary Table 6) for incidence risk of breast cancer.

## Discussion

Leveraged data from large UK Biobank cohort, the present study evaluated the associations between consumptions of three types of beverages and risk of breast cancer. We observed that consuming >1 serving/day of pure fruit/vegetable juice was associated with increased risk of breast cancer. However, we found no associations of SSB and ASB with breast cancer risk. These findings may provide insight into the association between beverage intake and the prevention of breast cancer.

In line with our study, several epidemiological studies observed an increased risk of breast cancer with consumption of pure fruit/vegetable juice. A meta-analysis including seven prospective studies showed that fruit juice consumption was associated with a higher breast cancer risk with risk ratio of 1.04 (12). In contrast, other studies reported no association between pure fruit/vegetable juice and breast cancer (8, 13–16). These inconsistent results may be due to the differences in exposure definitions or statistical power across studies. In our analysis, we observed a statistically significant trend between pure fruit/vegetable juice consumption and breast cancer risk. However, the association was non-significant when comparing the highest intake group to non-consumers. This indicates that, although the trend suggests a potential linear relationship, the effect estimate for the highest consumption category may be attenuated by insufficient statistical power resulting from a relatively small sample size in that group.

The biological mechanisms underlying this association remain unclear, but several potential explanations may account for this association. Pure fruit/vegetable juice is a kind of beverage that may extremely content a mass of sugar, particularly fructose. It was reported that pure fruit/vegetable juice, especially fruit juice, may contain even more fructose per unit than SSB (29). Accumulating epidemiological evidence suggests that high fructose intake may be positively associated with breast cancer risk (30, 31). Experimental studies also indicated that high levels of fructose intake may be linked to specific metabolic changes in the mammary gland and potentially promote breast carcinogenesis (32). Fructose is rapidly metabolized in the liver (33), bypassing the rate-limiting step of glycolysis and promoting the activation of carbohydrate-responsive element-binding protein (34), which may drive lipogenesis and glucose metabolism (35). These changes may contribute to hyperinsulinemia (36) and increased expression of insulin-like growth factor-1 (IGF-1) (37), both of which have been implicated in tumor proliferative, anti-apoptotic effects in the mammary gland, and activation of glucocorticoid receptors in tumors (38–41). It should also be noted that differences in the food matrix between fruit juices and whole fruits, as well as variations in their absorption by the body, may influence health outcomes. The beneficial compounds and nutrients in whole fruits, like vitamins and fiber, may help mitigate the adverse effects of sugar and offer protective benefits (42). Further research is needed to elucidate the multifactorial mechanisms underlying the relationship between pure fruit/vegetable juice and breast cancer.

In our main analyses, we did not observe significant associations between SSB intake and breast cancer risk. Although a sensitivity analysis using mutually exclusive beverage groups revealed a significant positive association between exclusive SSB consumption and breast cancer risk, the absence of this association in our primary pre-specified analysis precludes a definitive conclusion. Several cohort studies, including the Framingham Offspring Cohort (10), the Canadian Study of Diet, Lifestyle and Health (15), and the Nurses' Health Study (43), reported null findings regarding the association between SSB and breast cancer which aligns with the results of our main analysis. Whereas, several other large-scale cohort studies suggested a significant association between SSB consumption and breast cancer risk. Chazelas et al. found that consumption of sugary drinks was significantly associated with the risk of breast cancer using data on 79,724 participants (n_case_= 639) over median follow-up of 5.1years (8). Additionally, researchers from the Melbourne Collaborative Cohort Study (9) and the Seguimiento Universidad de Navarra cohort study (44) found an increased risk of post-menopausal breast cancer. The inconsistency in research findings may stem from variations in beverage consumption patterns across different time periods. The significant association observed in our mutually exclusive beverage analysis highlights the critical role of exposure contamination in nutritional epidemiology. Their collective effects likely attenuate SSB-specific risks through cancellation biases or additive confounding. Crucially, our results must be interpreted within the historical context of SSB consumption patterns. The dietary data in UK Biobank reflect intake levels recorded between 2006 and 2010—a period preceding the steep global rise in SSB consumption documented over the past quarter-century (6). Population-wide intake during this era was substantially lower than in contemporary cohorts; thus, even our mutually exclusive analysis likely captured a dose below the biological threshold required for detectable carcinogenesis in many individuals. Considering the conflicting results, more high-quality, long-term study is required to fully understand the associations of SSB and breast cancer.

In contrast to SSB, ASB contain few to no calories, making them a likely attractive substitute for SSB. In the present study, ASB intake showed a non-significant association with breast cancer risk, consistent with Chazelas's (8) and Romanos-Nanclares's (43) studies. Although the substitution analysis revealed a statistically significant association between ASB consumption and breast cancer risk, the effects of consumption of ASB on the risk of breast cancer warrant further investigation. ASB have been shown to stimulate sweet receptors, which can activate the cephalic phase insulin response (CPIR) and trigger the release of intestinal hormones (45). Additionally, their neurological effects may elicit a food-rewarding response in the brain (45). However, the UK Biobank does not specify the types of artificial sweeteners contained in ASB, which limits our ability to investigate their potential association with breast cancer incidence.

This study's strengths include its prospective design within the UK Biobank, featuring a large sample population and an extended follow-up period. Additionally, the diverse range of demographic, lifestyle, and other factors captured by the UK Biobank enabled more robust adjustments for potential confounders. Nevertheless, this study has certain limitations. Firstly, a key limitation of this study stems from the demographic characteristics of the UK Biobank cohort. As all participants were aged 40 years or older, our findings cannot be generalized to younger populations regarding beverage consumption and breast cancer risk. Additionally, the relatively healthier baseline status and higher socioeconomic background of UK Biobank participants may introduce selection bias through the “healthy volunteer effect.” Secondly, exposure was assessed via the 24-h dietary recall questionnaire, which may involve unavoidable measurement error and recall bias. However, in a sensitivity analysis excluding participants who self-reported weekly dietary changes, we obtained similar results, supporting the robustness of our findings. Finally, since this was an observational study, residual confounding and reverse causality were unavoidable. Over the 10-year follow-up maybe introduce exposure misclassification.

## Conclusion

In this large cohort study, consumption of pure fruit/vegetable juice was associated with an increased risk of breast cancer, suggesting that healthier beverage choices may play a role in breast cancer prevention. Additional studies are still needed to confirm the observed associations and explore potential mechanisms.

## Data Availability

Publicly available datasets were analyzed in this study. This data can be found here: https://www.ukbiobank.ac.uk UK Biobank Application Number 99685.
