# Supplementary material for: Association of sweetened beverage intake with incident risk of breast cancer: a prospective cohort study
Source: Front Nutr. 2026 Jan 14;12:1680542. doi: 10.3389/fnut.2025.1680542 (PMC12846961; doi:10.3389/fnut.2025.1680542)
Supplement: Supplementary file 1 [file Table_1.docx]

**Supplementary files**

**Supplementary Table 1**. Association of beverage intake with breast cancer risk after excluding participants developing events during first 2 years of follow-up.

**Supplementary Table 2**. Association of beverage intake with breast cancer risk mutually exclusive beverage groups.

**Supplementary Table 3**. Association of beverage intake with breast cancer risk excluding participants who self-reported weekly changes in daily diet.

**Supplementary Table 4**. Subgroup analyses for the associations between risk of incident breast cancer and sugar-sweetened beverage intake.

**Supplementary Table 5**. Subgroup analyses for the associations between risk of incident breast cancer and artificial sweetened beverage intake.

**Supplementary Table 6**. Subgroup analyses for the associations between risk of incident breast cancer and nature juice intake.

**Supplementary Table 1**. Association of beverage intake with breast cancer risk after excluding participants developing events during first 2 years of follow-up.

| **Beverages** | **Incident cases/Person years** | **Model 1** | |  | **Model 2** | |  | **Model 3** | |
| --- | --- | --- | --- | --- | --- | --- | --- | --- | --- |
|  |  | **HR (95% CI)** | ***P* value** |  | **HR (95% CI)** | ***P* value** |  | **HR (95% CI)** | ***P* value** |
| **Sugar-sweetened beverages** |  |  |  |  |  |  |  |  |  |
| 0 serving/day | 1324/571048.88 | Ref |  |  | Ref |  |  | Ref |  |
| >0 and ≤1 serving/day | 492/208114.36 | 1.03 (0.93-1.15) | 0.543 |  | 1.04 (0.94-1.16) | 0.430 |  | 1.05 (0.94-1.16) | 0.405 |
| >1 serving/day | 197/83019.55 | 1.07 (0.92-1.25) | 0.361 |  | 1.07 (0.92-1.25) | 0.375 |  | 1.10 (0.94-1.28) | 0.256 |
| *P* for trend |  | 0.310 |  |  | 0.278 |  |  | 0.200 |  |
| **Artificially sweetened beverages** | |  |  |  |  |  |  |  |  |
| 0 serving/day | 1563/668849.6 | Ref |  |  | Ref |  |  | Ref |  |
| >0 and ≤1 serving/day | 221/93004.31 | 1.04 (0.90-1.20) | 0.581 |  | 1.02 (0.88-1.18) | 0.787 |  | 1.02 (0.89-1.18) | 0.779 |
| >1 serving/day | 229/100328.87 | 1.03 (0.89-1.18) | 0.700 |  | 0.99 (0.86-1.14) | 0.850 |  | 0.99 (0.86-1.14) | 0.877 |
| *P* for trend |  | 0.592 |  |  | 0.938 |  |  | 0.965 |  |
| **Pure fruit/vegetable juice** |  |  |  |  |  |  |  |  |  |
| 0 serving/day | 954/431920.58 | Ref |  |  | Ref |  |  | Ref |  |
| >0 and ≤1 serving/day | 935/375856.35 | 1.12 (1.02-1.22) | 0.018 |  | 1.14 (1.04-1.25) | 0.006 |  | 1.14 (1.04-1.25) | 0.005 |
| >1 serving/day | 124/54405.86 | 1.03 (0.85-1.24) | 0.771 |  | 1.06 (0.87-1.27) | 0.574 |  | 1.09 (0.90-1.32) | 0.390 |
| *P* for trend |  | 0.087 |  |  | 0.033 |  |  | 0.019 |  |

Abbreviations: Ref, reference; HR, hazard ratio; CI, confidence interval.

Values are standardized to 1 serving (250 mL).

Model 1 was adjusted for age. Model 2 was additionally adjusted for ethnicity, education qualification, Townsend deprivation index, physical activity, smoking status, drinking status, body mass index, hypertension status, diabetes status, cardiovascular disease status and family history of breast cancer. Model 3 was additionally adjusted for total energy intake, total sugar intake and AHEI score, menopausal status, parity, hormone replacement therapy and oral contraceptive pill use.

**Supplementary Table 2**. Association of beverage intake with breast cancer risk mutually exclusive beverage groups.

| **Beverages** | **Incident cases/Person years** | **Model 1** | |  | **Model 2** | |  | **Model 3** | |
| --- | --- | --- | --- | --- | --- | --- | --- | --- | --- |
|  |  | **HR (95% CI)** | ***P* value** |  | **HR (95% CI)** | ***P* value** |  | **HR (95% CI)** | ***P* value** |
| **Sugar-sweetened beverages** | |  |  |  |  |  |  |  |  |
| 0 serving/day | 673/239694.36 | Ref |  |  | Ref |  |  | Ref |  |
| >0 and ≤1 serving/day | 171/58151.28 | 1.06 (0.90-1.26) | 0.488 |  | 1.07 (0.91-1.27) | 0.414 |  | 1.10 (0.93-1.31) | 0.268 |
| >1 serving/day | 101/31636.48 | 1.20 (0.97-1.48) | 0.090 |  | 1.19 (0.97-1.47) | 0.101 |  | 1.32 (1.05-1.64) | 0.016 |
| *P* for trend |  | 0.088 |  |  | 0.086 |  |  | 0.015 |  |
| **Artificially sweetened beverages** | |  |  |  |  |  |  |  |  |
| 0 serving/day | 673/239694.36 | Ref |  |  | Ref |  |  | Ref |  |
| >0 and ≤1 serving/day | 70/24321.73 | 1.05 (0.82-1.35) | 0.678 |  | 1.03 (0.81-1.32) | 0.800 |  | 1.04 (0.81-1.33) | 0.746 |
| >1 serving/day | 126/42565.55 | 1.12 (0.92-1.35) | 0.255 |  | 1.08 (0.89-1.31) | 0.452 |  | 1.09 (0.89-1.32) | 0.408 |
| *P* for trend |  | 0.242 |  |  | 0.444 |  |  | 0.394 |  |
| **Pure fruit/vegetable juice** |  |  |  |  |  |  |  |  |  |
| 0 serving/day | 673/239694.36 | Ref |  |  | Ref |  |  | Ref |  |
| >0 and ≤1 serving/day | 640/189338.58 | 1.19 (1.07-1.33) | 0.002 |  | 1.21 (1.08-1.35) | 0.001 |  | 1.23 (1.10-1.37) | <0.001 |
| >1 serving/day | 87/29274.31 | 1.06 (0.85-1.32) | 0.611 |  | 1.09 (0.87-1.36) | 0.452 |  | 1.16 (0.92-1.46) | 0.218 |
| *P* for trend |  | 0.019 |  |  | 0.009 |  |  | 0.002 |  |

Abbreviations: HR, hazard ratio; CI, confidence interval.

Values are standardized to 1 serving (250 mL). Model 1 was adjusted for age and another two beverages. Model 2 was additionally adjusted for ethnicity, education qualification, Townsend deprivation index, physical activity, smoking status, drinking status, body mass index, hypertension status, diabetes status, cardiovascular disease status and family history of breast cancer. Model 3 was additionally adjusted for total energy intake, total sugar intake and AHEI score, menopausal status, parity, hormone replacement therapy and oral contraceptive pill use.

**Supplementary Table 3**. Association of beverage intake with breast cancer risk excluding participants self-reported weekly changes in daily diet.

| **Beverages** | **Incident cases/Person years** | **Model 1** | |  | **Model 2** | |  | **Model 3** | |
| --- | --- | --- | --- | --- | --- | --- | --- | --- | --- |
|  |  | **HR (95% CI)** | ***P* value** |  | **HR (95% CI)** | ***P* value** |  | **HR (95% CI)** | ***P* value** |
| **Sugar-sweetened beverages** | |  |  |  |  |  |  |  |  |
| 0 serving/day | 1602/529264.90 | Ref |  |  | Ref |  |  | Ref |  |
| >0 and ≤1 serving/day | 603/192550.20 | 1.05 (0.95-1.15) | 0.334 |  | 1.06 (0.96-1.16) | 0.246 |  | 1.06 (0.96-1.17) | 0.226 |
| >1 serving/day | 230/75822.39 | 1.05 (0.91-1.20) | 0.504 |  | 1.05 (0.91-1.21) | 0.495 |  | 1.07 (0.93-1.24) | 0.345 |
| *P* for trend |  | 0.316 |  |  | 0.266 |  |  | 0.185 |  |
| **Artificially sweetened beverages** | |  |  |  |  |  |  |  |  |
| 0 serving/day | 1901/620164.70 | Ref |  |  | Ref |  |  | Ref |  |
| >0 and ≤1 serving/day | 270/85582.72 | 1.05 (0.93-1.19) | 0.439 |  | 1.03 (0.90-1.17) | 0.687 |  | 1.03 (0.90-1.17) | 0.705 |
| >1 serving/day | 264/91890.10 | 0.86 (0.86-1.12) | 0.808 |  | 0.94 (0.82-1.07) | 0.357 |  | 0.94 (0.82-1.07) | 0.356 |
| *P* for trend |  | 0.957 |  |  | 0.493 |  |  | 0.487 |  |
| **Pure fruit/vegetable juice** |  |  |  |  |  |  |  |  |  |
| 0 serving/day | 1146/397937.95 | Ref |  |  | Ref |  |  | Ref |  |
| >0 and ≤1 serving/day | 1135/349620.59 | 1.12 (1.03-1.21) | 0.009 |  | 1.14 (1.05-1.23) | 0.003 |  | 1.15 (1.05-1.25) | 0.002 |
| >1 serving/day | 154/50078.97 | 1.06 (0.90-1.26) | 0.472 |  | 1.10 (0.93-1.30) | 0.288 |  | 1.13 (0.96-1.36) | 0.146 |
| *P* for trend |  | 0.032 |  |  | 0.009 |  |  | 0.004 |  |

Abbreviations: HR, hazard ratio; CI, confidence interval.

Values are standardized to 1 serving (250 mL). Model 1 was adjusted for age. Model 2 was additionally adjusted for ethnicity, education qualification, Townsend deprivation index, physical activity, smoking status, drinking status, body mass index, hypertension status, diabetes status, cardiovascular disease status and family history of breast cancer. Model 3 was additionally adjusted for total energy intake, total sugar intake and AHEI score, menopausal status, parity, hormone replacement therapy and oral contraceptive pill use.

**Supplementary Table 4**. Subgroup analyses for the associations between risk of incident breast cancer and sugar-sweetened beverage intake.

| Subgroup | Sugar-sweetened beverages | HR (95%CI) | *P* for interaction |
| --- | --- | --- | --- |
| **Age** |  |  |  |
| Age <60 years | 0 serving/d | Ref | 0.147 |
|  | >0-1 serving/d | 1.09 (0.98-1.21) |  |
|  | >1 serving/d | 0.98 (0.84-1.14) |  |
| Age ≥60 years | 0 serving/d | Ref |  |
|  | >0-1 serving/d | 0.96 (0.84-1.09) |  |
|  | >1 serving/d | 1.08 (0.88-1.32) |  |
| **BMI** |  |  |  |
| BMI <25kg/m^2^ | 0 serving/d | Ref | 0.069 |
|  | >0-1 serving/d | 1.05 (0.93-1.19) |  |
|  | >1 serving/d | 1.03 (0.99-1.44) |  |
| BMI 25-30kg/m^2^ | 0 serving/d | Ref |  |
|  | >0-1 serving/d | 1.05 (0.92-1.19) |  |
|  | >1 serving/d | 0.87 (0.70-1.21) |  |
| BMI ≥30kg/m^2^ | 0 serving/d | Ref |  |
|  | >0-1 serving/d | 0.98 (0.82-1.18) |  |
|  | >1 serving/d | 0.94 (0.73-1.21) |  |
| **Type 2 diabetes** |  |  |  |
| Diabetes status (-) | 0 serving/d | Ref | 0.921 |
|  | >0-1 serving/d | 1.03 (0.95-1.12) |  |
|  | >1 serving/d | 1.02 (0.90-1.16) |  |
| Diabetes status (+) | 0 serving/d | Ref |  |
|  | >0-1 serving/d | 1.02 (0.72-1.43) |  |
|  | >1 serving/d | 0.85 (0.51-1.41) |  |
| **Physical activity** |  |  |  |
| Low physical activity | 0 serving/d | Ref | 0.653 |
|  | >0-1 serving/d | 1.07 (0.87-1.32) |  |
|  | >1 serving/d | 1.22 (0.91-1.64) |  |
| Moderate physical activity | 0 serving/d | Ref |  |
|  | >0-1 serving/d | 1.07 (0.94-1.22) |  |
|  | >1 serving/d | 1.00 (0.81-1.22) |  |
| High physical activity | 0 serving/d | Ref |  |
|  | >0-1 serving/d | 1.01 (0.87-1.17) |  |
|  | >1 serving/d | 1.03 (0.83-1.30) | 0.967 |
| **Menopausal status** |  |  |  |
| Premenopausal | 0 serving/d | Ref |  |
|  | >0-1 serving/d | 1.00 (0.87-1.16) |  |
|  | >1 serving/d | 1.08 (0.89-1.31) |  |
| Postmenopausal | 0 serving/d | Ref |  |
|  | >0-1 serving/d | 1.05 (0.95-1.15) |  |
|  | >1 serving/d | 0.97 (0.82-1.14) |  |

Abbreviations: Ref, reference; HR, hazard ratio; CI, confidence interval.

Models were calculated based on fully adjusted model, adjusted for age, ethnicity, education qualification, Townsend deprivation index, physical activity, smoking status, drinking status, BMI, hypertension status, diabetes status, cardiovascular disease status and family history of breast cancer, total energy intake, total sugar intake, AHEI score, menopausal status, parity, hormone replacement therapy and oral contraceptive pill use.

**Supplementary Table 5**. Subgroup analyses for the associations between risk of incident breast cancer and artificial sweetened beverage intake.

| Subgroup | Artificially sweetened beverages | HR (95%CI) | *P* for interaction |
| --- | --- | --- | --- |
| **Age** |  |  |  |
| Age <60 years | 0 serving/d | Ref | 0.093 |
|  | >0-1 serving/d | 0.96 (0.83-1.10) |  |
|  | >1 serving/d | 0.99 (0.87-1.13) |  |
| Age ≥60 years | 0 serving/d | Ref |  |
|  | >0-1 serving/d | 1.12 (0.95-1.33) |  |
|  | >1 serving/d | 0.84 (0.68-1.03) |  |
| **BMI** |  |  |  |
| BMI <25kg/m^2^ | 0 serving/d | Ref | 0.085 |
|  | >0-1 serving/d | 1.06 (0.88-1.27) |  |
|  | >1 serving/d | 0.93 (0.75-1.15) |  |
| BMI 25-30kg/m^2^ | 0 serving/d | Ref |  |
|  | >0-1 serving/d | 1.12 (0.95-1.32) |  |
|  | >1 serving/d | 0.83 (0.69-1.00) |  |
| BMI ≥30kg/m^2^ | 0 serving/d | Ref |  |
|  | >0-1 serving/d | 0.81 (0.64-1.03) |  |
|  | >1 serving/d | 1.10 (0.91-1.32) |  |
| **Type 2 diabetes** |  |  |  |
| Diabetes status (-) | 0 serving/d | Ref | 0.958 |
|  | >0-1 serving/d | 1.02 (0.91-1.14) |  |
|  | >1 serving/d | 0.95 (0.84-1.06) |  |
| Diabetes status (+) | 0 serving/d | Ref |  |
|  | >0-1 serving/d | 0.94 (0.61-1.46) |  |
|  | >1 serving/d | 0.93 (0.65-1.35) |  |
| **Physical activity** |  |  |  |
| Low physical activity | 0 serving/d | Ref | 0.357 |
|  | >0-1 serving/d | 1.11 (0.85-1.44) |  |
|  | >1 serving/d | 1.15 (0.90-1.48) |  |
| Moderate physical activity | 0 serving/d | Ref |  |
|  | >0-1 serving/d | 0.96 (0.80-1.14) |  |
|  | >1 serving/d | 0.79 (0.65-0.96) |  |
| High physical activity | 0 serving/d | Ref |  |
|  | >0-1 serving/d | 1.00 (0.81-1.23) |  |
|  | >1 serving/d | 1.02 (0.84-1.24) | 0.932 |
| **Menopausal status** |  |  |  |
| Premenopausal | 0 serving/d | Ref |  |
|  | >0-1 serving/d | 0.98 (0.82-1.18) |  |
|  | >1 serving/d | 1.00 (0.85-1.18) |  |
| Postmenopausal | 0 serving/d | Ref |  |
|  | >0-1 serving/d | 1.04 (0.91-1.19) |  |
|  | >1 serving/d | 0.91 (0.79-1.05) |  |

Abbreviations: Ref, reference; HR, hazard ratio; CI, confidence interval.

Models were calculated based on fully adjusted model, adjusted for age, ethnicity, education qualification, Townsend deprivation index, physical activity, smoking status, drinking status, BMI, hypertension status, diabetes status, cardiovascular disease status and family history of breast cancer, total energy intake, total sugar intake, AHEI score, menopausal status, parity, hormone replacement therapy and oral contraceptive pill use.

**Supplementary Table 6**. Subgroup analyses for the associations between risk of incident breast cancer and nature juice intake.

| Subgroup | Pure fruit/vegetable juice | HR (95%CI) | *P* for interaction |
| --- | --- | --- | --- |
| **Age** |  |  |  |
| Age <60 years | 0 serving/d | Ref | 0.372 |
|  | >0-1 serving/d | 1.19 (1.08-1.30) |  |
|  | >1 serving/d | 1.15 (0.94-1.40) |  |
| Age ≥60 years | 0 serving/d | Ref |  |
|  | >0-1 serving/d | 1.05 (0.94-1.17) |  |
|  | >1 serving/d | 1.12 (0.90-1.40) |  |
| **BMI** |  |  |  |
| BMI <25kg/m^2^ | 0 serving/d | Ref | 0.286 |
|  | >0-1 serving/d | 1.21 (1.09-1.36) |  |
|  | >1 serving/d | 1.15 (0.92-1.45) |  |
| BMI 25-30kg/m^2^ | 0 serving/d | Ref |  |
|  | >0-1 serving/d | 1.03 (0.92-1.16) |  |
|  | >1 serving/d | 1.08 (0.84-1.38) |  |
| BMI ≥30kg/m^2^ | 0 serving/d | Ref |  |
|  | >0-1 serving/d | 1.15 (0.98-1.34) |  |
|  | >1 serving/d | 1.21 (0.88-1.68) |  |
| **Type 2 diabetes** |  |  |  |
| Diabetes status (-) | 0 serving/d | Ref | 0.628 |
|  | >0-1 serving/d | 1.14 (1.06-1.22) |  |
|  | >1 serving/d | 1.15 (0.99-1.33) |  |
| Diabetes status (+) | 0 serving/d | Ref |  |
|  | >0-1 serving/d | 0.97 (0.71-1.31) |  |
|  | >1 serving/d | 1.04 (0.54-2.00) |  |
| **Physical activity** |  |  |  |
| Low physical activity | 0 serving/d | Ref | 0.506 |
|  | >0-1 serving/d | 1.26 (1.05-1.52) |  |
|  | >1 serving/d | 1.30 (0.91-1.88) |  |
| Moderate physical activity | 0 serving/d | Ref |  |
|  | >0-1 serving/d | 1.05 (0.94-1.18) |  |
|  | >1 serving/d | 1.08 (0.85-1.37) |  |
| High physical activity | 0 serving/d | Ref |  |
|  | >0-1 serving/d | 1.22 (1.07-1.40) |  |
|  | >1 serving/d | 1.18 (0.90-1.55) | 0.229 |
| **Menopausal status** |  |  |  |
| Premenopausal | 0 serving/d | Ref |  |
|  | >0-1 serving/d | 1.08 (0.95-1.22) |  |
|  | >1 serving/d | 0.88 (0.67-1.17) |  |
| Postmenopausal | 0 serving/d | Ref |  |
|  | >0-1 serving/d | 1.15 (1.05-1.25) |  |
|  | >1 serving/d | 1.27 (1.07-1.51) |  |

Abbreviations: Ref, reference; HR, hazard ratio; CI, confidence interval. Models were calculated based on fully adjusted model, adjusted for age, ethnicity, education qualification, Townsend deprivation index, physical activity, smoking status, drinking status, BMI, hypertension status, diabetes status, cardiovascular disease status and family history of breast cancer, total energy intake, total sugar intake, AHEI score, menopausal status, parity, hormone replacement therapy and oral contraceptive pill use.
